# Ten years on: Ongoing challenges in screening for cryptococcal antigenaemia in people with advanced HIV disease in South Africa

**DOI:** 10.4102/sajhivmed.v27i1.1788

**Published:** 2026-07-31

**Authors:** Rhys Wenlock, Emily Prendergast, Kyla Murphy, Tshiamo Mmotsa, David Lawrence, Rudzani Mashau, Joseph Jarvis, Thomas Harrison, Síle Molloy, Nelesh Govender

**Affiliations:** 1 Department of Infectious Diseases and Tropical Medicine, Newcastle-upon-Tyne Hospitals NHS Foundation Trust, Newcastle, United Kingdom; 2 Wits Mycology Division, School of Pathology, University of the Witwatersrand, Johannesburg, South Africa; 3 CIDRI Africa, Institute of Infectious Diseases and Molecular Medicine, University of Cape Town, Cape Town, South Africa; 4 Clinical Research Department, Faculty of Infectious and Tropical Diseases, London School of Hygiene and Tropical Medicine, London, United Kingdom; 5 Botswana Harvard AIDS Institute Partnership, Gaborone, Botswana; 6 Research Centre for Infection and Immunity, St. George’s University of London, London, United Kingdom

**Keywords:** advanced HIV disease, cryptococcal disease, screening, South Africa, health systems

## Abstract

Screening for early asymptomatic cryptococcal antigenaemia is a crucial step in preventing symptomatic cryptococcal meningitis and reducing mortality among people with advanced HIV disease. However, even when early cryptococcal disease is detected, a significant number of people with advanced HIV disease still develop meningitis and die, suggesting barriers to care following a positive serum cryptococcal antigen result. Ten years after the initiation of a national screening programme in South Africa, we discuss progress and implementation challenges and highlight potential solutions identified through a clinical trial to reduce barriers to care for people with early cryptococcal disease. Analysing systems that care for individuals with early cryptococcal disease and creating interventions to optimise available resources are key steps in reducing HIV-associated cryptococcal disease mortality worldwide.

**What this study adds:** By studying barriers to screening for cryptococcal disease, interventions can be designed to strengthen care pathways for people with advanced HIV disease and to sustain the gains of screening programmes in the face of constrained resources.

## Introduction

*Cryptococcus* causes meningitis that is the second leading cause of death in people with advanced HIV disease (AHD, defined as CD4 count <200 cells/μL, or WHO clinical stage 3 or 4), accounting for one-fifth of HIV-related deaths worldwide.^1^ The majority of these deaths (73%) occur in Africa. An estimated 4.4% of people with AHD have cryptococcal antigenaemia, that is, the presence of cryptococcal antigen (CrAg) in the blood. Untreated, there is a high rate of progression to cryptococcal disease with increasing mortality risk.^2,3,4^ At the earliest stage of infection patients do not experience meningitis symptoms, as CrAg is present in the blood but absent from the cerebrospinal fluid (CSF). Studies demonstrate that pre-emptively treating these patients with fluconazole prevents progression to meningitis and reduces mortality.^2^ If antigenaemia is inadequately untreated, CrAg may become detected in the CSF, yet patients may remain asymptomatic because of low fungal burden. Evidence suggests that up to one third of people with asymptomatic cryptococcal antigenaemia have a positive CSF CrAg when tested,^5^ and that patients with asymptomatic cryptococcal meningitis (CM) have milder disease and lower acute mortality than patients with symptomatic CM directly attending hospital.^3^ If asymptomatic cryptococcal disease remains untreated, people with AHD will eventually progress to develop symptomatic CM at a median duration of 22–43 days.^6,7^

Given that each stage of cryptococcal disease is associated with increased mortality risk and long-term disability, and that prevalence among people living with HIV is inversely related to CD4 count, early identification and treatment of cryptococcal disease among people with AHD is key to preventing CM-related morbidity and mortality. A reflex screening programme initiated in 2016 in South Africa at all CD4 testing laboratories addresses this priority by automatically testing all individuals with a CD4 count below 100 cells/μL (200 cells/μL in the Western Cape) for CrAg, regardless of symptoms. CrAg is detected using a lateral flow assay, which has a rapid turnaround time and 100% negative predictive value for subsequent development of CM, if antiretroviral therapy (ART) is started promptly.^8^ Laboratory-based reflex screening was adopted as a national strategy over provider-initiated models, as it reduces the number of visits required for at-risk individuals, and a cost-effectiveness analysis demonstrated that it was cost-saving.^9^

Lumbar puncture is recommended for all people with AHD with a positive serum CrAg.^10^ If the CSF sample is negative for CrAg, at least 1 year of oral fluconazole is recommended to prevent progression to CM, which can be started alongside ART. All patients with a positive CSF CrAg, whether asymptomatic or symptomatic, are recommended to receive inpatient meningitis treatment with a single high dose (10 mg/kg) of liposomal amphotericin B, and 14 days of flucytosine (100 mg/kg per day) and fluconazole (1200 mg/daily for adults).^11^ Following this induction phase, fluconazole 800 mg/daily for 8 weeks then 200 mg/daily for a total duration of at least 1 year and until their CD4 is above 200 cells/μL and their viral load is suppressed.

Healthcare workers can receive reflex CrAg results by looking up individual results on the National Health Laboratory Service (NHLS) database (TrakCare) or registering for weekly CrAg results-for-action emails to access line-lists of people with a positive reflex CrAg result who need urgent follow-up. The strength of the reflex programme lies in high coverage. Since 2016, 99% of 2 364 173 CD4 samples with counts below 100 cells/μL underwent reflex CrAg testing, with 123 815 testing positive. In the Western Cape, where CrAg screening includes people with CD4 counts < 200 cells/μL, an additional 72 871 people were tested, with 1965 testing positive.

Although cryptococcal disease detection has increased since the introduction of the reflex screening programme, it is unclear whether the intervention has decreased CM incidence and mortality. This is likely a result of multiple factors. Firstly, reflex screening relies on CD4 testing, which has significantly declined since the beginning of the programme.^12^ Secondly, shortcomings of the reflex programme and persistent barriers to timely, effective care mean that screened patients may not experience clinical benefit, as suggested by evidence demonstrating similar in-hospital mortality of patients both with and without recent CrAg screening, and higher mortality than patients included in clinical trials.^13^ Thirdly, even when screening and pre-emptive antifungal treatment are implemented, mortality remains higher in people with AHD with cryptococcal antigenaemia (18% – 24%) than those without cryptococcal antigenaemia (9% – 15%).^2^ A significant proportion of this mortality is because of cryptococcal disease^14^; in an investigation of the causes of death following CrAg screening and antifungal treatment in South Africa, cryptococcal disease was still a contributing or causative factor in 71% of deaths. Therefore, clinical trials are investigating the impact of combination antifungal treatment on mortality among patients with cryptococcal antigenaemia compared to single-agent therapy.^15^

Here, we discuss barriers to cryptococcal disease screening in South Africa and propose opportunities for improving its impact.

## Discussion

### Decline in CD4 testing

**FIGURE 1:**
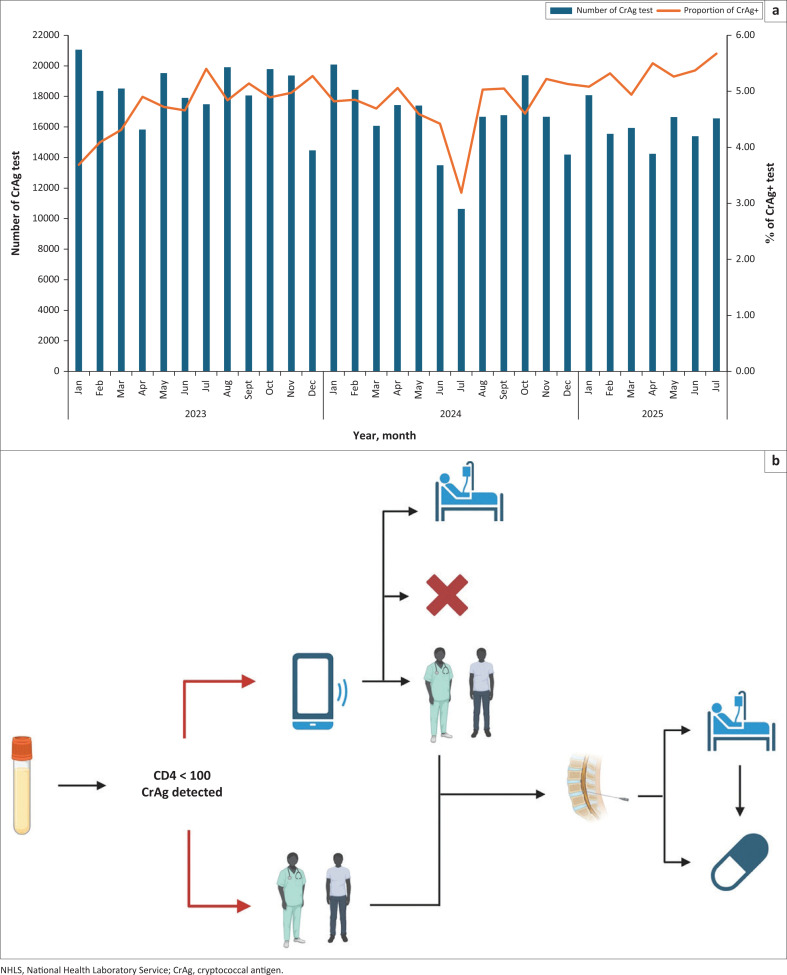
(a) The impact of the NHLS ransomware attack on detection of cryptococcal disease in South Africa (2024); and (b) the pathway to care for patients with cryptococcal antigenaemia in South Africa.

As the reflex programme automatically tests CrAg from remnant CD4 samples, it only detects cryptococcal disease in patients who receive a CD4 test. Therefore, if CD4 tests are not widely and regularly conducted, cases of cryptococcal disease will be missed. Over the past decade, CD4 testing has declined because of a combination of programmatic constraints, the widespread adoption of HIV test-and-treat (compared to CD4-based thresholds) and, more recently, the withdrawal of United States Agency for International Development and US President’s Emergency Plan for AIDS Relief funding. The dependence on CD4 testing was clearly illustrated during the 40-day NHLS ransomware attack in 2024. During this period, the inability to upload test requests and results to the NHLS laboratory information management system, coupled with severe operational pressures on laboratory staff, resulted in a reduction in CD4 testing.^16^ This coincided with a marked decrease in the number of positive reflex CrAg results detected (Figure 1).

### Limitations of the reflex screening programme in South Africa

Only 11% of people receive a lumbar puncture following a positive CrAg result in South Africa.^17,18^ Furthermore, a high proportion of people (48%) do not return for follow-up, and fluconazole therapy is delayed, inadequately prescribed, or not administered in those who do, particularly ambulatory patients with asymptomatic disease.^17,19^

A disadvantage of adopting a reflex-testing approach is that people with AHD and healthcare providers are disconnected from the screening process, rendering them passive rather than active participants.^9^ As CrAg is reflexively tested in the laboratory following detection of a low CD4 count, the requesting clinician may remain unaware of the result until the next appointment which is often over 7 days following the initial visit, by which time the patient could have developed CM and/or died.^20^ Studies in Kenya and Zimbabwe support this by demonstrating that centralised laboratory testing leads to delayed feedback of results compared to point-of-care testing. People with HIV also highlight the benefit of prompt results to guide treatment.^21^

These delays are particularly problematic for people with asymptomatic cryptococcal antigenaemia, as healthcare staff are not prompted by symptoms to urgently follow up results and patients may not seek healthcare until they develop symptoms. National uptake of CrAg results-for-action registration is low, with only 210 active subscriptions out of a nursing workforce of over 250 000.^22^ Without widespread awareness and use of notification systems such as the results-for-action list, laboratory reflex CrAg testing will remain disconnected from clinical sites, leading to missed CrAg results.

Surveillance metrics offer an opportunity to scrutinise the reflex screening programme. Whilst the NHLS CrAg dashboard already reports laboratory metrics (e.g. percentage of all CrAg tests with a CrAg-positive result in South Africa), downstream data of clinical outcomes (e.g. proportion of patients with a positive CrAg test who undergo lumbar puncture) are not currently reported, highlighting a crucial gap in surveillance. Lumbar puncture uptake could be extracted from laboratory data by triangulating positive blood CrAg tests with CSF samples on TrakCare: a recent analysis of 2166 positive plasma CrAg samples in South Africa found that only 10% had a corresponding logged CSF sample within 28 days.^18^ Methods to monitor treatment and outcomes following a positive CrAg result remain more difficult, as they rely on paper-based systems at clinics and hospitals.

### Pathway to care after recognition of a positive cryptococcal antigen result

If the responsible clinic identifies the positive reflex CrAg result, they should contact the patient, arrange a lumbar puncture, and initiate appropriate treatment.^10^ In under-resourced healthcare systems, clinicians manage large numbers of patients and people with AHD, who are often severely unwell, difficult to contact, and difficult to engage because of intersecting factors such as limited healthcare education, stigma, immigration status, and low socioeconomic status.^23^ For example, in a qualitative methods study assessing a CrAg screening programme in Uganda, clinics reported difficulties contacting patients because of incorrect contact information and staff not having enough airtime to call patients.^24^ These challenges lead to poor follow-up, as demonstrated by the South African CAST-NET study, which found that among a cohort of 1651 patients with a positive CrAg test, only 39% had a documented review of meningitis symptoms at a median of 5 days after CrAg blood draw.^17^

If a healthcare worker succeeds in contacting the patient, concerns surrounding lumbar puncture, such as fear of death and paralysis, may deter them from attending the clinic or hospital. In a qualitative methods study of the perceptions of individuals with CM in Botswana and Uganda, there was widespread perception that lumbar punctures directly led to death.^25^ One reason cited by participants for consenting to lumbar puncture, despite perceived risks, was their physical condition and severe state of illness. Moreover, a qualitative methods study in Zambia demonstrated that healthcare workers also perceived that lumbar punctures were harmful to patients, with 28% reporting that it results in poor clinical outcomes and 60% reporting that it could cause paralysis.^26^ As patients with early cryptococcal disease have no meningitis symptoms, and less information is publicly available about asymptomatic disease, affected individuals may be more likely to delay or refuse lumbar puncture.

Some people with AHD may not be offered a lumbar puncture because of lack of healthcare worker training and awareness of the need for a lumbar puncture and oral fluconazole for asymptomatic patients. Barriers to healthcare workers performing lumbar punctures identified by a study in Zambia included limited lumbar puncture knowledge and expertise, time constraints, and fear of blame for adverse outcomes. Community engagement improves lumbar puncture uptake, yet patient education materials with clear messaging are often not widely available.^27^

If the patient consents to a lumbar puncture, the procedure may be delayed, or they may opt out because of delays in referral, transport, and clinical review, as they are often performed in under-resourced state hospitals in South Africa. If the lumbar puncture is performed, and the CSF CrAg is negative, a 14-day course of 1200 mg fluconazole daily is initiated as an outpatient, followed by 800 mg daily for 2 months and then 200 mg daily for at least a year and until the CD4 is > 200 cells/μL and HIV viral load is suppressed. Accurate prescriptions at each stage, patient counselling regarding different doses and course lengths, and close follow-up are crucial to successfully treat early cryptococcal disease and prevent progression to meningitis. However, given that screening, contact, investigation, and follow-up are split between the lab, clinics, and hospitals, it may be difficult to deliver clear and consistent communication about treatment to the patient, leading to insufficient or lack of treatment (Figure 1). For example, among a cohort of people with cryptococcal antigenaemia between 2017 and 2019 in South Africa, only 32% were dispensed an adequate dose of fluconazole.^17^

### Clinical trials investigating dual antifungal therapy for cryptococcal antigenaemia

People with AHD and cryptococcal antigenaemia continue to have markedly higher mortality than those without cryptococcal antigenaemia, which may indicate a need for enhanced pre-emptive antifungal treatment. The Single dose Liposomal Amphotericin for Asymptomatic Cryptococcal Antigenemia Trial (ACACIA)^15^ is a randomised controlled trial investigating superiority of a single-dose of intravenous liposomal amphotericin B followed by oral fluconazole versus oral fluconazole monotherapy on 6-month CM-free survival in people with cryptococcal antigenaemia in Uganda. An efficacy analysis in a subgroup of trial participants with low baseline serum CrAg titres (≤ 1:80) did not find a survival benefit in the intervention arm, though the analysis was under-powered. The trial continues to assess the same dual antifungal regimen in a high-titre group (≥ 1:160). EFFECT (ISRCTN30579828) is an ongoing phase 3 randomised controlled trial comparing the efficacy of combining flucytosine with fluconazole versus fluconazole alone on all-cause 6-month mortality in adults with asymptomatic cryptococcal antigenaemia without CSF involvement in South Africa and Tanzania.

### Learning from research: Opportunities to analyse and alleviate barriers to care for patients with cryptococcal disease

Beyond its direct aims, the EFFECT trial has provided an opportunity to study the barriers to streamlined and effective care for this population as trial site teams identify and closely follow up patients in partnership with the public healthcare systems. By having more time, resources, and in-depth knowledge of the risks associated with cryptococcal antigenaemia, EFFECT trial investigators raise awareness of early disease and directly improve patient management. On closure of the trial, it will be important to ensure the longevity of its impact by creating systems that address the gaps identified in care.

CAST-OFF (Cryptococcal Antigenaemia Screening and Treatment – Observations from the Field) is a sub-study of the EFFECT trial which will use mixed methods to analyse the pathway to care for patients with asymptomatic early cryptococcal disease. Focus group discussions with clinic staff linked to EFFECT trial sites in Gauteng, KwaZulu-Natal, the Eastern Cape and the Western Cape provinces will explore the timeline between a patient’s reflex CrAg test and antifungal treatment and follow-up. Questionnaires completed by clinic nurses and doctors will elucidate gaps in knowledge of early cryptococcal disease and highlight where educational initiatives should be targeted. Interviews with EFFECT investigators will help to understand the impact of the trial on screening and treatment, shortcomings of the screening programme, use of CrAg results-for-action reports, and problems encountered with contacting patients and arranging appropriate management. These insights will be complemented by NHLS data comparing system metrics between EFFECT trial sites before and after trial implementation. The findings of CAST-OFF will inform targeted interventions to improve the notification system of reflex CrAg results, bridge the gaps between laboratories, patients in communities, clinics, and hospitals, and raise awareness of early cryptococcal disease.

Enhanced surveillance of clinical outcomes is also required to complement CAST-OFF findings and to identify priority areas for intervention, with proposed metrics outlined in Box 1. Although establishing and maintaining comprehensive surveillance systems poses substantial challenges, South Africa can leverage both the infrastructure and lessons of the NICD’s GERMS-SA (Group for Enteric, Respiratory and Meningeal disease Surveillance in South Africa) sentinel hospital surveillance platform to inform future programme development. The critical challenge ahead will be extending this surveillance into community and primary care settings to a degree not yet achieved outside short-term cohort studies.

BOX 1 Proposed surveillance metrics for people with cryptococcal disease in South Africa.

1. Proportion of people with AHD who undergo CrAg screening
2. Proportion of people with AHD and a CrAg test who have new cryptococcal antigenaemia
3. Proportion of people with new cryptococcal antigenaemia who undergo lumbar puncture to exclude meningitis
4. Proportion of people with new cryptococcal antigenaemia but no meningitis who receive pre-emptive fluconazole
5. Proportion of people with new cryptococcal antigenaemia who start or restart antiretroviral therapy
6. Proportion of people with CM who receive combination antifungal treatment
7. Proportion of people with CM who start or restart antiretroviral therapy within a month following antifungal treatment
8. Proportion of people with CM who die in hospital

CM, cryptococcal meningitis; AHD, advanced HIV disease; CrAg, cryptococcal antigen.

A CD4 test is recommended for anyone newly diagnosed with HIV or re-entering care. If the CD4 count is below 100 cells/μL (or 200 cells/μL in the Western Cape), the laboratory reflexively tests the sample for CrAg. The red arrows indicate potential significant delay during the following step where the positive result is either noted by a healthcare professional at the patient’s next appointment or via electronic systems (Trakcare or results-for-action reports), hard copies of results sent to their facility, or a telephone call from the laboratory. The healthcare professional must arrange a lumbar puncture for anyone with a positive result, yet some patients may already have developed symptomatic meningitis or died, and others may not be contactable. If the patient undergoes a lumbar puncture, and CrAg is not detected in the CSF, they should be prescribed at least 1 year of oral fluconazole. If CrAg is detected in the CSF, they must receive treatment for meningitis, followed by a course of at least 1 year of oral fluconazole.

## Conclusion

The full impact of antifungal treatment and CrAg screening programmes in preventing cryptococcal deaths will only be realised if individuals are diagnosed promptly, receive treatment quickly, and are closely followed up. Implementation of the EFFECT trial has highlighted gaps in the current screening programme but also provided an opportunity to study and begin to overcome the barriers to streamlined and effective care for those with early cryptococcal disease. An evidence-based approach, incorporating the findings of the EFFECT trial and CAST-OFF, complemented by enhanced surveillance and community engagement and education, is required to ensure that effective and robust solutions are implemented to reduce the morbidity and mortality associated with cryptococcal disease.
